# Transient expression analysis of synthetic promoters containing F and D *cis*-acting elements in response to *Ascochyta rabiei* and two plant defense hormones

**DOI:** 10.1186/s13568-019-0919-x

**Published:** 2019-12-04

**Authors:** Farhad Shokouhifar, Marjan Bahrabadi, Abdolreza Bagheri, Mojtaba Mamarabadi

**Affiliations:** 1 Research Center for Plant Sciences, Ferdowsi University of Mashhad, Mashhad, Iran; 2 Department of Biotechnology and Plant Breeding, Faculty of Agriculture, Ferdowsi University of Mashhad, Mashahad, Iran; 3 Department of Plant Protection, Faculty of Agriculture, Ferdowsi University of Mashhad, Mashahad, Iran

**Keywords:** Pathogen-inducible promoter, *Agrobacterium*-mediated transient expression, Elicitor, Chickpea, Necrotrophic pathogens

## Abstract

Introduction of a foreign gene coding for a pathogen resistant protein into the target plant and constitutive expression of Resistance (R) proteins may confer high level of resistance. However, genetic engineering could lead to reprogramming of molecular mechanisms that manage physiological behavior, which in turn could lead to undesired results. Therefore, using a pathogen-inducible synthetic promoter approach, response to pathogens could be more specific. *Ascochyta rabiei* is a destructive fungal pathogen in chickpea production. In this study, we analyzed the expression pattern of three synthetic promoters in response to pathogen and two defense hormones. We have tested three synthetic pathogen-inducible promoters designated as (1) synthetic promoter-D box-D box (SP-DD), (2) synthetic promoter-F element-F element (SP-FF) and (3) synthetic promoter-F element-F element-D box-D box (SP-FFDD) via *Agrobacterium* transient expression assay. The *cis*-acting element designated as ‘D’ is a 31 base pair sequence from the promoter of parsley pathogenesis-related gene 2 (*PR2* gene) and the *cis*-acting element designated as ‘F’ is a 39 base pairs sequence from the promoter of *Arabidopsis AtCMPG1* gene. We used mycelial extracts from two pathotypes of *A. rabiei* as elicitor to define the responsiveness of the promoters against pathogen. Plant phytohormones including salicylic acid and methyl jasmonate were also used to study the promoter sensitivity in plant signaling pathways. Our results showed that the SP-FF promoter was highly inducible to *A. rabiei* and methyl jasmonate as well, while the SP-DD promoter was more sensitive to salicylic acid. The SP-FFDD promoter was equally responsive to both pathotypes of *A. rabiei* which is probably due to the complex nature of box D *cis*-acting element.

## Introduction

Chickpea (*Cicer arietinum* L.) is the third most significant, compared to beans and peas, cool season legume in the world and its seed have been consumed by humans since about 7000 years before Christ. It has been used as a main protein source for the poor communities in many parts of the semi-arid tropical regions in Africa and Asia. The most important goals of chickpea breeding programs are to promote genetic potential for increasing cultivar production and also elimination of the disease effects and environmental stresses (Singh 1997). Ascochyta blight is a major disease of chickpea across the world which is caused by the fungus *Ascochyta rabiei* (Homrich et al. 2008) Labrousse (teleomorph, *Didymella rabiei* (Kovachevski) v. Arx.) and often leads to high yield losses. It has been believed that the presence of sexual recombination in the population of this fungus donates its population genetic diversity (Chongo et al. 2004). Limited resistance in the current chickpea cultivars can easily be broken down due to the high variable forms of this pathogen. Instead, there are a broad spectrum range of resistance genes in different plants which potentially can be used for transformation of legume cultivars.

One of the most important steps in gene transferring strategy is the regulation of transgene expression and the task of a suitable promoter in a host plant. Different studies have shown that the constitutive expression of the resistance genes often resulted in poor quality plants (Gurr and Rushton 2005; Hammond-Kosack and Parker 2003).

The approach of synthetic pathogen-inducible promoter is a suitable alternative for the other transgene expression methods which aims only crop resistant to pathogens and simultaneously reduced the crop yields. An ideal pathogen inducible promoter would only be activated in response to target pathogens. Furthermore, it should be able to express the transgene both locally and temporarily. Some promoter components like *cis*-regulatory elements are used in construction of synthetic promoters which provide kind of high flexibility in the measurement of expression quantity and also type of inducibility. *Agrobacterium*-mediated transient expression system is an appropriate method for analyzing synthetic promoter strength by a short time application of biotic and abiotic treatments after agro-injection (Liu et al. 2011; Yang et al. 2000).

In the present study, three synthetic pathogen inducible promoters named; synthetic promoter-D box-D box (SP-DD), synthetic promoter-F element-F element (SP-FF) and synthetic promoter-F element-F element-D box-D box (SP-FFDD) were analyzed in response to *Ascochyta rabiei* mycelial extracts. These pathogen inducible promoters have already been constructed and reported in the other publication (Shokouhifar et al. 2011a). The analyzed regulatory segments were as follows: (1) SP-DD, with the sequence of 5′-TAC AAT TCA AAC ATT GTT CAA ACA AGG AAC CTC TAG TTA CAA TTC AAA CAT TGT TCA AAC AAG GAA-3′, containing two copies of box D originated from parsley pathogenesis-related gene 2 (*PR2* gene) with 31 base pairs length without any clarified core boxes (Rushton et al. 2002); (2) SP-FF, with the sequence of 5′-TGC ATT CGA CTA GTT TGT CAA TGT CAT TAA ATT CAA ACA TTC AAC GGT CAA TTT CTA GAG CCC TTC-3′, containing two copies of box F originated from *Arabidopsis thaliana AtCMPG1* gene promoter with 39 base pairs length and three GTCA core sequences (Heise et al. 2002) and (3) SP-FFDD with the sequence of 5′-TTG TCA ATG TCA TTA AAT TCA AAC ATT CAA CGG TCA ATT TCT AGT TTG TCA ATG TCA TTA AAT TCA AAC ATT CAA CGG TCA ATT TCT AGT TAC AAT TCA AAC ATT GTT CAA ACA AGG AAC CTC TAG TTA CAA TTC AAA CAT TGT TCA AAC AAG GAA CCT CTA G-3′, which is a combination of these boxes upstream of *Cauliflower mosaic virus* (CaMV) 35S minimal promoter. They were individually fused with an intron-containing ß-glucuronidase reporter gene and transferred to tobacco leaves (*Nicotiana tabacum* cv. Xanthi and *Nicotiana benthamiana*) using agro-injection technique. Both boxes have previously shown high inducibility in response to the necrotrophic pathogens (Rushton et al. 2002; Shokouhifar et al. 2011a). The promoter function was evaluated in response to the elicitors of the phytopathogen fungus *Ascochyta rabiei* and two plant defense hormones; salicylic acid and methyl jasmonate. The results showed that, the induction of SP-DD synthetic promoter was higher in response to salicylic acid compared to the treatments by methyl jasmonate and *Ascochyta rabiei* elicitors. The SP-FF synthetic promoter was highly inducible to methyl jasmonate treatment as well as both elicitors of *Ascochyta rabiei* specially isolate number 009 (ASR009). The SP-FFDD promoter showed an inducibility to both elicitors and plant defense signaling hormones.

## Materials and methods

### Biological resources

Tobacco plants, plasmids and Agrobacterium strain LBA4404 were provided by the Plant Research Institute, Ferdowsi University of Mashhad. *Ascochyta rabiei* pathotypes were provided by the Microorganisms Collection of Ferdowsi University of Mashhad (WDCM 1207), Iran. Tobacco plants including *Nicotiana tabacum* cv. Xanthi and *Nicotiana benthamiana* were grown in a greenhouse at 22 °C and 16:8 h light/dark photoperiod and used at the age of 8 weeks. *Agrobacterium tumefaciens* strain LBA4404 was used for plant transformation. The plasmid constructs applied for promoter analysis were pGDD (containing two copies of D *cis*-acting elements + CaMV 35S minimal promoter upstream of an intron-containing ß-glucuronidase reporter gene), pGFF (containing two copies of F *cis*-acting elements + CaMV 35S minimal promoter upstream of an intron-containing ß-glucuronidase reporter gene) and pGFFDD (containing two copies of both D and F *cis*-acting elements + CaMV 35S minimal promoter upstream of an intron-containing ß-glucuronidase reporter gene), respectively (Shokouhifar et al. 2011a). The *Agrobacterium* strain LBA4404 containing pGCGi plasmid construct (including CaMV 35S complete promoter upstream of an intron-containing ß-glucuronidase reporter gene) (Shokouhifar et al. 2014) was used as a positive control. Routine procedures were performed for competent cell preparation, plasmid transformation, polymerase chain reaction (PCR), agarose gel production and electrophoresis of bacterial strain LBA4404 (Sambrook and Russell 2001; Weigel and Glazebrook 2005). The transformed colonies were confirmed by colony PCR method using specific forward and reverse primers named PSh4-F (5′-TCC TTT AGC AGC CCT TGC GC-3′) and PSh4-R (5′-CGA TCC AGA CTG AAT GCC CAC A-3′), respectively (Shokouhifar et al. 2014).

The confirmation assay for eukaryotic expression was performed with separate cultivation of transformed colonies (containing pGCGi, pGDD, pGFF and pGFFDD) in β-glucuronidase (GUS) staining solution. *Agrobacterium tumefaciens* harboring pBI121 vector (including CaMV 35S complete promoter upstream of ß-glucuronidase reporter gene) (Chen et al. 2003) was used as a positive prokaryotic control. All cultivated colonies were kept at 37 °C overnight.

Cell preparation procedure was performed by cultivation of confirmed colonies in Luria–Bertani (LB) liquid medium containing rifampicin and kanamycin as antibiotics. Cultured media were incubated at 28 °C with constant shaking (150 rpm) for 72 h. Bacterial cells were settled by centrifugation at 2500 rpm and suspended in induction medium (1 g l^−1^ NH_4_Cl, 0.3 g l^−1^ MgSO_4_·7H_2_O, 0.015 g l^−1^ of KCl, 0.01 g l^−1^ CaCl_2_, 2.5 mg l^−1^ FeSO_4_·7H_2_O, 2 mM phosphate buffer, 20 mM MES, 1% sucrose, pH: 5.5) overnight. The cells were then recollected by centrifugation and diluted in the injection medium containing MgSO4 and MES at ph: 5.5 to obtain a final OD_600_ of 0.8 for the plant injection (Yang et al. 2000).

The resulted bacterial suspensions were individually injected into the plants via agro-injection method. The cells of *Agrobacterium* strain LBA4404 containing pGCGi construct were independently prepared for plant injection using the same method.

### Agro-injection

Agro-injection step was performed on the nearly complete expanded tobacco leaves of *N. tabacum* cv. Xanthi and *Nicotiana benthamiana* using of 1 ml syringes without needles. Bacterial cell suspensions were injected into the space among plant cells while they were still attached to the plant. Each bacterial suspension containing individual plasmid construct was separately injected into the back side of different plant leaves with two repetitions. Then, the whole plants were covered with plastic bags and kept in a germinator at 22 °C under 16 and 8 h light and darkness, respectively. The plastic bags were removed in the next day and the plants were remained at the same conditions at least for 2 days.

### Salicylic acid and methyl jasmonate treatments

48 h after agro-injection (Liu et al. 2011; Yang et al. 2000), 2 mM of salicylic acid and 50 μM of methyl jasmonate (Shokouhifar et al. 2011a) both from Sigma Aldrich (Darmstadt, Germany) were separately sprayed on agro-injected leaves for each tobacco species. Two tobacco plants from *N. tabacum* cv. Xanthi and *Nicotiana benthamiana* species were used without any agro-injection as salicylic acid control. Two plants were also selected for methyl jasmonate control. The control plants were separately sprayed with salicylic acid and methyl jasmonate. Leaf disks were sampled for GUS assay 24 h after treatment with salicylic acid and methyl jasmonate (Yang et al. 2000). Leaf discs imaging was carried out after histochemical staining and chlorophyll removal procedures using a digital microscope (Dino-Lite Am-313 model T Plus made in Taiwan).

### Total fungal elicitor preparation

Two pathotypes of *Ascochyta rabiei* with the following strain/code number; FUM 1003/ASR003 (Pathotype No. 3) and FUM 1006/ASR009 (Pathotype No. 6) were obtained from the Microorganisms Collection of Ferdowsi University of Mashhad (WDCM 1207), Iran. They had been classified in our previous studies by (Shokouhifar et al. 2003b). The fungal pathotypes were cultured on PDA (Potato Dextrose Agar, Merck, Germany) to prepare fresh mycelium. Two plugs of the fresh mycelium were cut and transferred to 100 ml PDB medium (Potato Dextrose Broth, Merck, Germany) in 500 ml Erlenmeyer flask and incubated at 24 ± 2 °C with 120 rpm for 10 days. The Fungal mycelium were collected and washed for three times by distilled water and transferred to 100 ml of 200 mM phosphate buffer (pH = 7.00). Collected mycelium were homogenized using a homogenizer apparatus (Heidolph DIAX 900, Germany) at 4 °C for 5 min. Mycelium debris were precipitated by 15 min centrifugation in 10,000 rpm at 4 °C. Supernatant were aliquot into 10 ml tubes and stored at − 80 °C.

### Fungal elicitor treatments

24 h after agro-injection the tobacco leaves were separately sprayed with 2 ml of mycelium extracts. The fungal extracts applied on the plant leaves (*N. tabacum* cv. Xanthi and *Nicotiana benthamiana*) in three replications. Four agro-injected leaves with different constructs were used for a single fungal pathotype treatment in each plant and this step was repeated for both tobacco species. From each tobacco species, two agro-injected plants were used as control without any fungal treatment. The leaf discs were sampled for GUS assay 48 h after injection that was equal to 24 h after fungal elicitor treatments.

## Results

### Transformation and colony PCR confirmation

**Fig. 1:**
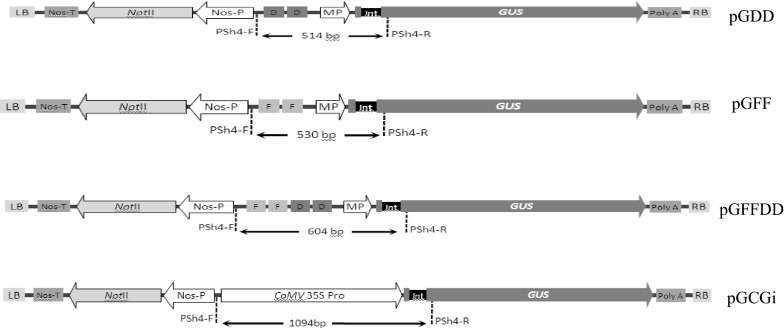
Schematic representation of T-DNA region in constructed synthetic promoters. pGDD, pGFF, and pGFFDD contain two repetitions of D element, two repetitions of F element and two repetitions of F and D elements which are located upstream of CaMV 35S minimal promoters, respectively. LB: left border, Nos-T: nopaline synthase terminator, *Npt*II: neomycin phosphotransferase II, Nos-P: nopaline synthase promoter, FF: dimer of the F *cis*-acting element, DD: dimer of the D *cis*-acting element, MP: sequence of − 46 to + 8 from CaMV 35S promoter as minimal promoter, GUS: β**-**glucuronidase containing intron, Poly A: poly adenylation tail, RB: right border. A pGCGi construct contains CaMV 35S promoter used as positive control

The activity and inducibility of three synthetic promoters contain pathogen-responsive elements F (Heise et al. 2002; Shokouhifar et al. 2011b) and D (Rushton et al. 2002) individually and in combination with each other, were investigated using agro-injection method (Kapila et al. 1997; Van der Hoorn et al. 2000; Yang et al. 2000) in the model plants; *Nicotiana tabacum* cv. Xanthi and *Nicotiana benthamiana*. The inducible effect of two *Ascochyta rabiei* pathotypes (ASR003 and ASR009) (Shokouhifar et al. 2003a, b) and two plant defense signaling hormones including salicylic acid and methyl jasmonate were evaluated using *Agrobacterium*-mediated transient expression system. For this purpose, following transformed vectors; pGFF, pGDD and pGFFDD containing the dimer forms of F and D elements individually and in a combination with each other were cloned upstream of CaMV 35S minimal promoter (− 46 to + 8, containing the TATA box). Then, they were fused with an intron containing ß-glucuronidase reporter gene and transferred to *Agrobacterium tumefaciens* LBA4404 (Weigel and Glazebrook 2005). The expression of these synthetic promoters were compared to the pGCGi vector (Shokouhifar et al. 2014) as a positive control. The pGCGi vector was contained a complete sequence of CaMV 35S promoter located upstream of GUS reporter gene (Fig. 1).

**Fig. 2:**
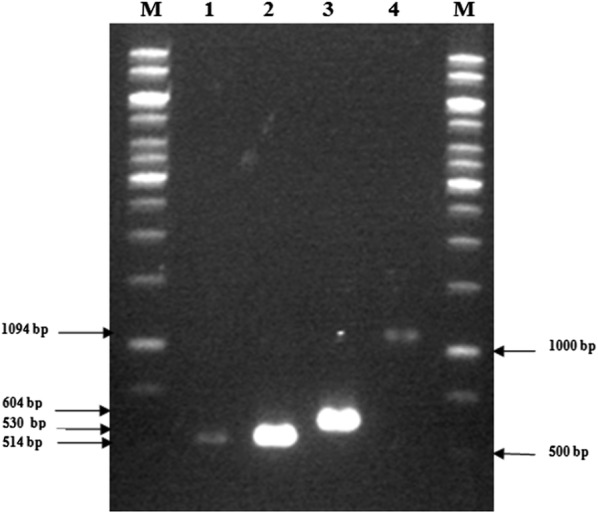
PCR confirmation of the transformed LBA4404 colonies containing; pGDD, pGFF, pGFFDD and pGCGi constructs. The line numbers 1, 2, 3 and 4 are amplified PCR product with specific primers PSh4-F/R related to the transformed *Agrobacterium* containing pGDD (514 bp), pGFF (530), pGFFDD (604 bp) and pGCGi (1094 bp), respectively, M is 1 kb DNA size marker

The occurrence of transformation in *Agrobacterium* cells was confirmed by colony-PCR method using PSh4-F/R specific primers. The electrophoresis pattern of PCR products showed the specific bands for the constructs in expected sizes (Fig. 2).

### Confirmation of eukaryotic gene expression

**Fig. 3:**
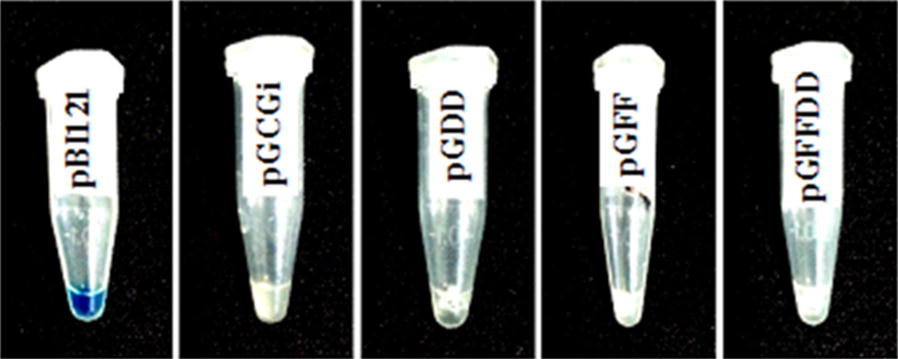
Lack of prokaryotic expression in pGCGi, pGDD, pGFF, and pGFFDD constructs compared to the prokaryotic expression in pBI121 vector

Lack of mRNA splicing in prokaryotes means that, they cannot express intron-containing genes. As a result, adding of an intron to GUS reporter gene, prevents *Agrobacterium* cells to express detectable GUS activity while eukaryotes like plants can efficiently splice mRNA to produce translational competent transcripts (Cazzonelli and Velten 2003; Ohta et al. 1990). In order to demonstrate the absence of prokaryotic expression, the confirmed colonies containing each construct (pGFF, pGDD, pGFFDD and pGCGi) were cultivated on LB kanamycin medium and they subsequently were used in GUS histochemical staining assay procedures. As all constructs carrying the intron-containing GUS gene, pBI121 vector (Chen et al. 2003) was used as a positive control to check the assay reliability. The lack of intron in the *Agrobacterium* cells containing pBI121 vector resulted expression of ß-glucuronidase reporter gene in histochemical staining assay. Due to the presence of intron in GUS reporter gene in the other four constructs, no detectable prokaryotic GUS expression was observed on them (Fig. 3).

### Expression pattern of the synthetic promoters in response to plant hormones

Plants depending on their pathogen life styles are able to manage different defense pathways (Robert-Seilaniantz et al. 2011). In order to investigate the inducibility of the synthetic promoters, four LBA4404 colonies harboring pGFF, pGDD, pGFFDD and pGCGi constructs were injected into tobacco plants (*Nicotiana tabacum* cv. Xanthi and *Nicotiana benthamiana*) using agro-injection technique and their GUS activity have been analyzed. To elucidate the response of the synthetic promoters to plant defense signaling pathways, the effects of salicylic acid and methyl jasmonate were also evaluated on the GUS gene expression under control conditions.

### Salicylic acid (SA)

**Fig. 4:**
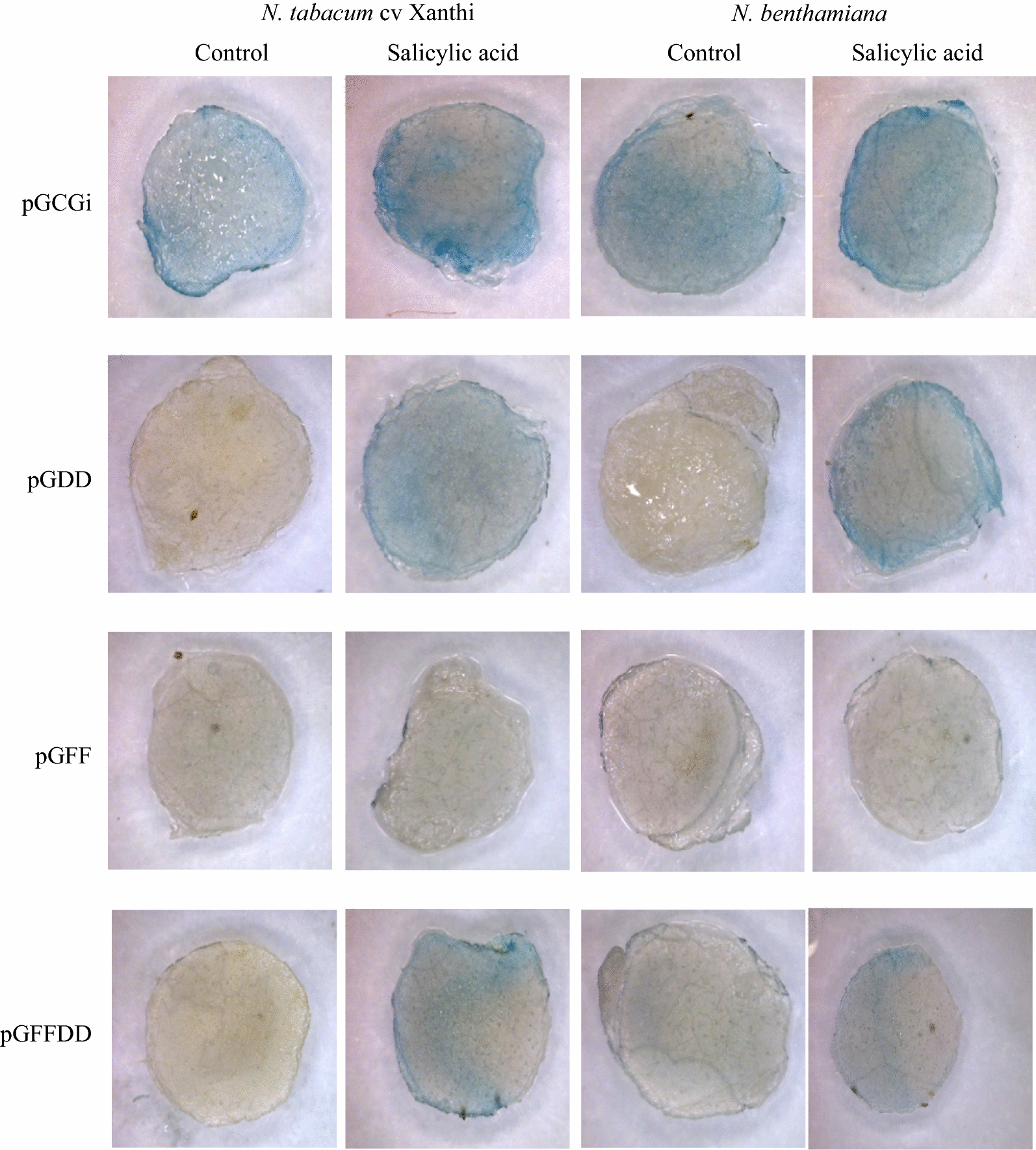
Effects of salicylic acid treatment on pGCGi, pGDD, pGFF and pGFFDD constructs evaluated on two tobacco species; *N. tabacum* cv. Xanthi and *N. benthamiana*. Agro-injected plants without salicylic acid treatment were used as control (More replications have been presented in Additional file 1: Fig. S1 attached to the paper)

The direct effect of salicylic acid as a phytohormone was assayed on the agro-injected leaves that transiently harboring the synthetic promoters. GUS activity was evaluated after 24 h. The longer treatment duo to the presence of cell wall degrading enzyme and several phytotoxins may produce necrotic cells in the leaf tissue which can interfere GUS assessment. pGCGi construct was used as a positive control. The results showed, lack of basal expression in agro- injected leaves by SP-FF, SP-DD and SP-FFDD promoters (Fig. 4). Moreover, response to 2 mM of salicylic acid has been observed for SP-DD and SP-FFDD promoters in both tobacco species (*Nicotiana tabacum* cv. Xanthi and *Nicotiana benthamiana*). GUS expression was more induced by SP-DD promoter than SP-FFDD promoter in response to salicylic acid. However, lack of any macroscopically predictable blue color in SP-FF promoter on treated leaves with salicylic acid suggests that, SP-FF promoter is not responsive to salicylic acid (Fig. 4).

### Methyl jasmonate (MJ)

**Fig. 5:**
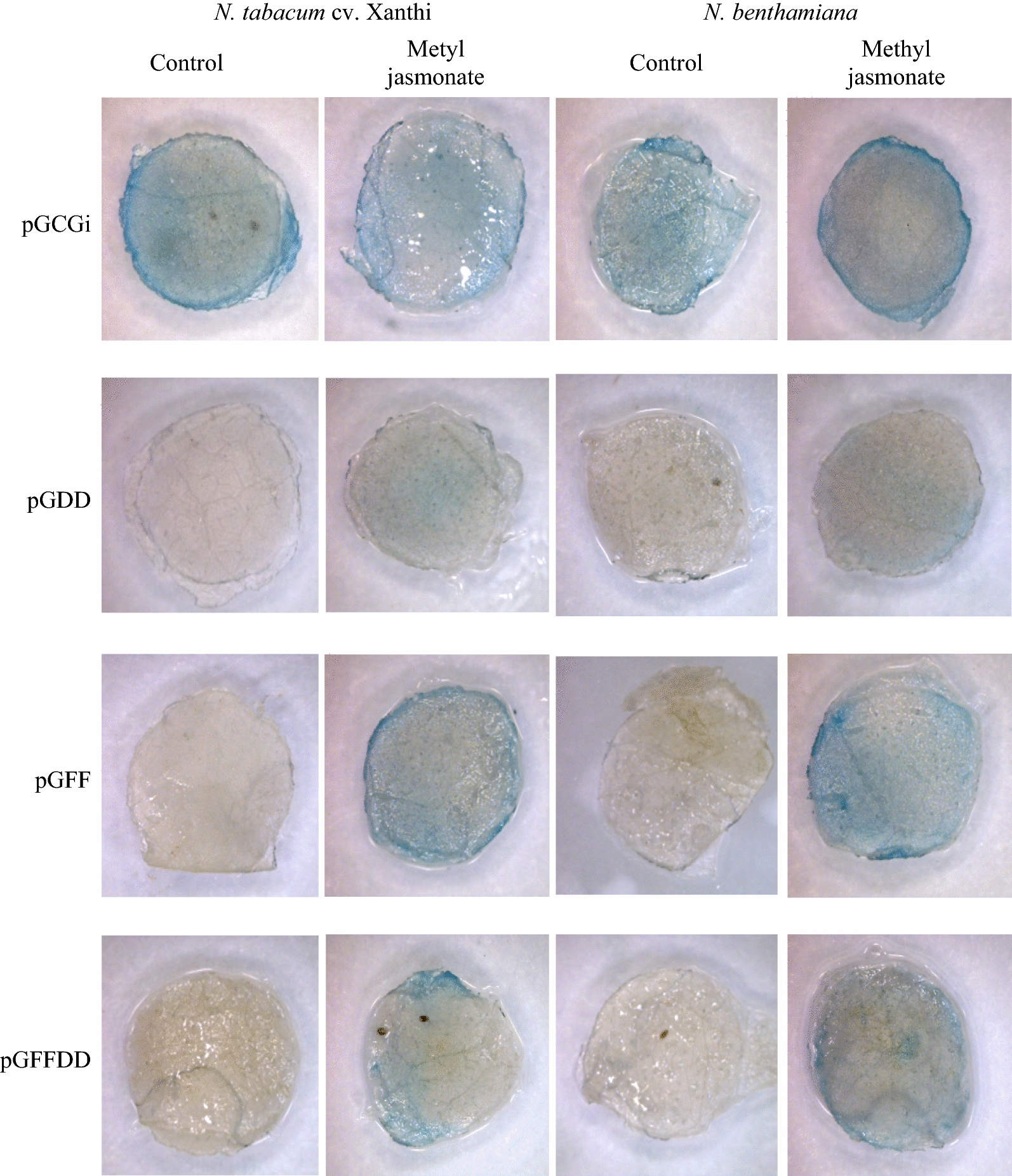
Effect of methyl jasmonate treatment on pGCGi, pGDD, pGFF and pGFFDD constructs evaluated on two tobacco species; *N. tabacum* cv. Xanthi and *N. benthamiana*. Agro-injected plants without methyl jasmonate treatment were used as control (More replications have been presented in Additional file 1: Fig. S2 attached to the paper)

Plants defend themselves against necrotrophic pathogens via jasmonic acid and ethylene dependent signaling pathways (Fujita et al. 2006; Kunkel and Brooks 2002; Pieterse and van Loon 1999). To reveal the efficiency of promoters against necrotrophic pathogens, agro-injected leaves were exposed to 50 μM of methyl jasmonate (a naturally occurring derivative of jasmonic acid) and the expression of reporter gene were assayed on the leaf disks 24 h after incubation (Liu et al. 2011). The results showed that GUS activity was obviously triggered by methyl jasmonate treatment in the injected plants by SP-FF promoter compared to untreated plants. In contrast, the SP-DD promoter was induced a little after methyl jasmonate treatment. Moderate amount of GUS expression was observed in SP-FFDD promoter compare to the other constructs. In addition, no detectable basal expression was detected in the SP-DD, SP-FF and SP-FFDD promoters compared to pGCGi promoter (Fig. 5).

### Effect of *Ascochyta rabiei*’s elicitors

**Fig. 6:**
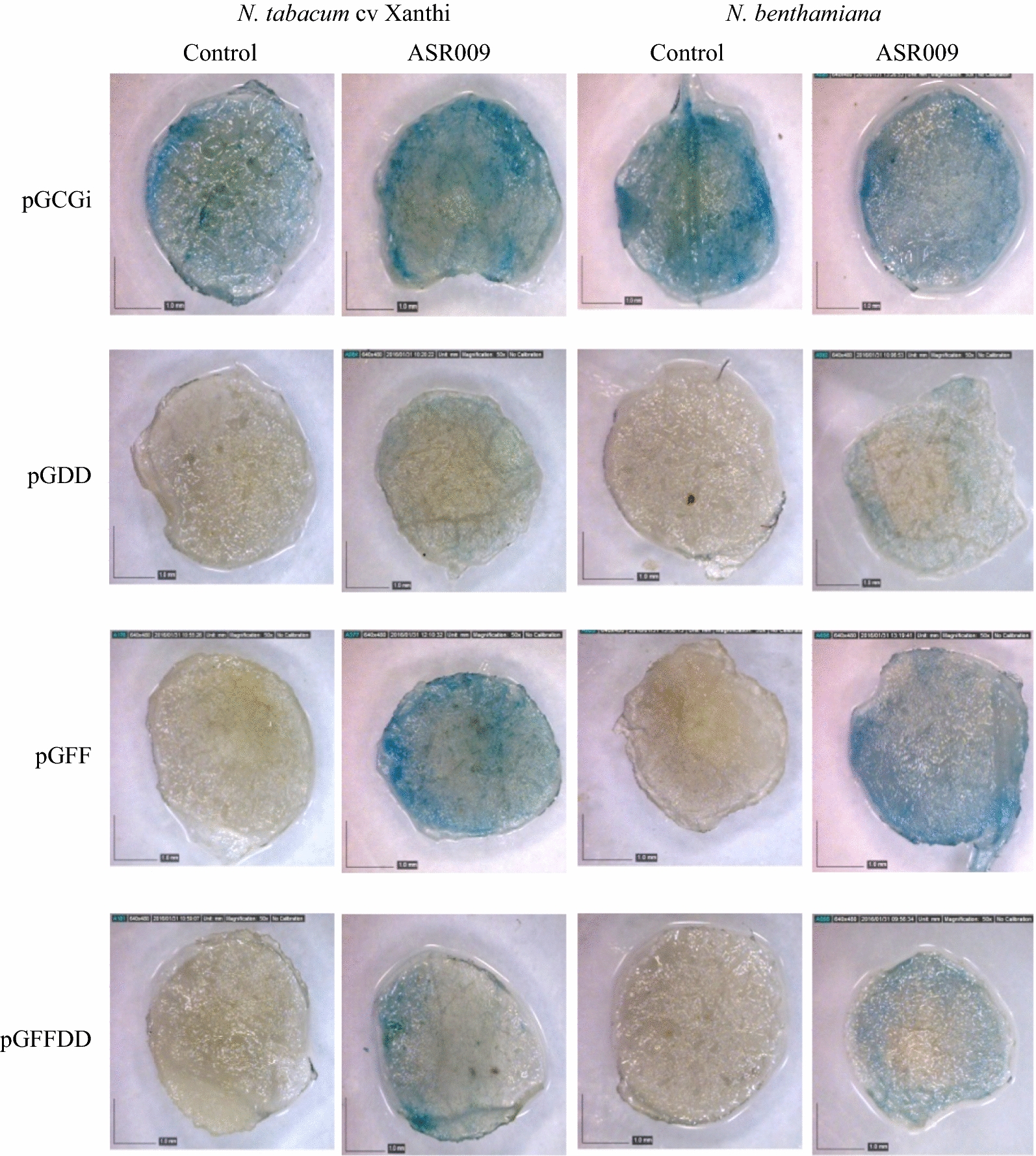
Effect of *Ascochyta rabiei* pathotype ASR009 on pGCGi, pGDD, pGFF and pGFFDD constructs evaluated on two tobacco species; *N. tabacum* cv. Xanthi and *N. benthamiana*. Agro-injected plants without treatment by fungal elicitor used as control (More replications have been presented in Additional file 1: Fig. S3 attached to the paper)

The effect of two *Ascochyta rabiei*’s elicitors named ASR003 and ASR009 was investigated on the expression of synthetic promoter reporter gene to elucidate the responsiveness of synthetic promoters to the fungal pathogens. The agro-injected plants with constructs have shown almost the same GUS expression pattern in response to the both elicitors. The amount of GUS activity in the leaves which were transiently harboring SP-FF promoter and treated with ASR009 pathotype was clearly more than GUS activity in the injected leaves with two other promoters and in non-treated plants as well. In addition, the visible GUS activity in SP-FFDD was higher than that of SP-DD in both tobacco species; *N. tabacum* cv. Xanthi and *Nicotiana benthamiana* (Fig. 6).

**Fig. 7:**
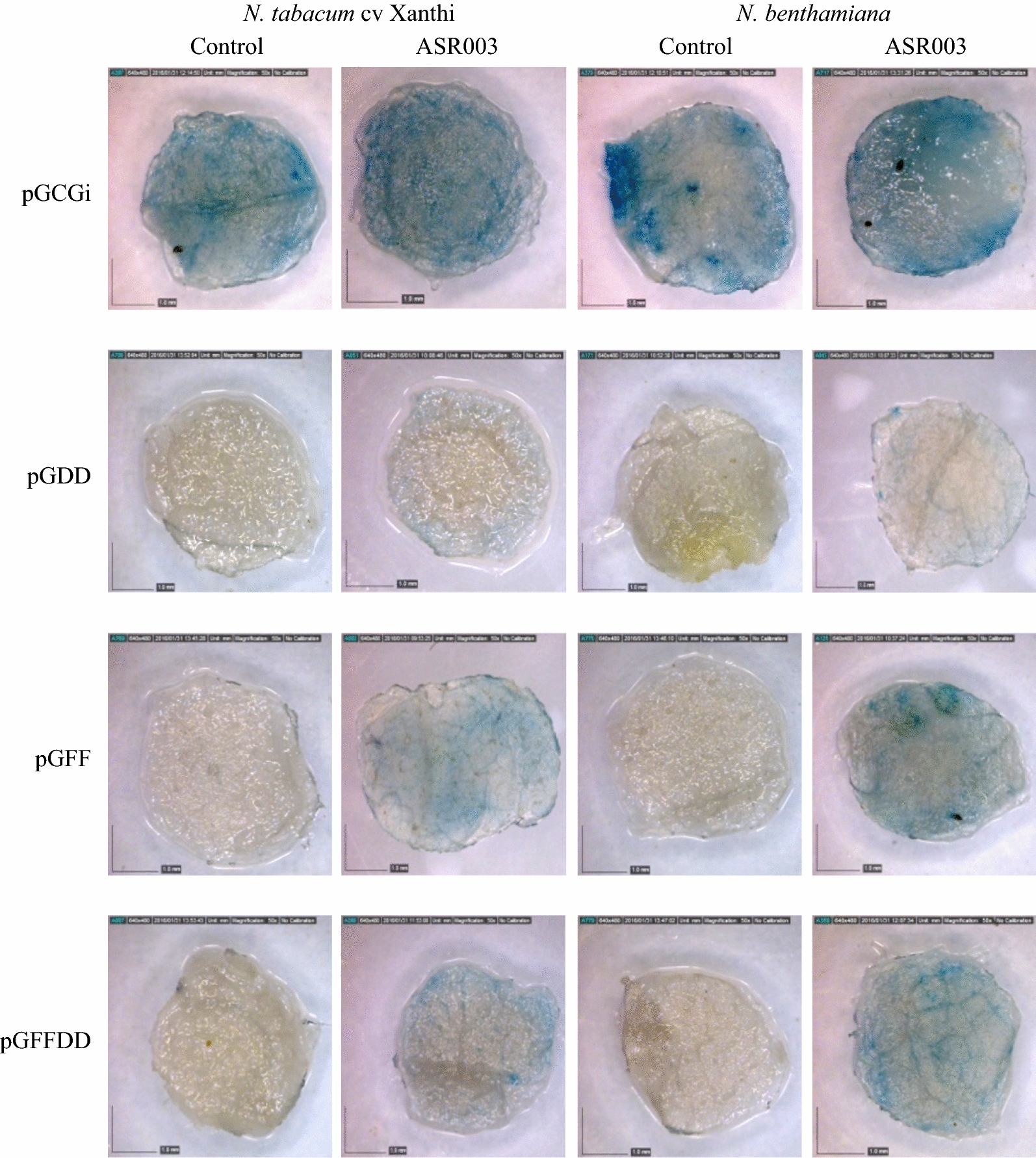
Effect of *Ascochyta rabiei* pathotype ASR003 on pGCGi, pGDD, pGFF and pGFFDD constructs evaluated on two tobacco species; *N. tabacum* cv. Xanthi and *N. benthamiana*. Agro-injected plants without treatment by fungal elicitor used as control (More replications have been presented in Additional file 1: Fig. S4 attached to the paper)

Similarly, the standing promoter inside pGFF construct induced more GUS activity than that of inside pGDD and pGFFDD constructs after treatment with ASR003 pathotype but the difference between pGFF and pGFFDD was not noticeable (Fig. 7).

## Discussions

This study was performed to examine the usability of *cis*-acting elements F and D in response to the plant phytohormones salicylic acid and methyl jasmonate and two elicitors of necrotrophic fungus *A. rabiei* (ASR003 and ASR009). The constructs were previously made and the functional analysis of one of them (pGFF) was evaluated via *Agrobacterium*-mediated stable transformation (Shokouhifar et al. 2011a). An efficient *Agrobacterium*-mediated transient expression method was used for analyzing promoter sensitivity. This method has frequently been applied in the other researches as well (Raikwar et al. 2015; Srivastava et al. 2014; Wu et al. 2014).

The number of *cis*-acting elements and their order in combination are two important issues in construction of a synthetic promoter (Gurr and Rushton 2005; Rushton et al. 2002). The selection of two copies for D and F *cis*-acting element was based on this fact that, increasing the number of each *cis*-acting element copies will increase the promoter strength. However, two copies had the best inducibility and additional copies for each element increased the background expression (Rushton et al. 2002). Moreover, characteristics of F and D *cis*-acting elements were examined in previous researches and the obtained results showed that these elements are pathogen inducible and not wound inducible (Heise et al. 2002; Rushton et al. 2002). In the present study, the response of dimer form of F (FF) and D (DD) elements individually and in combination with both elements (FFDD) to some pathotypes of *A. rabiei* as a necrotrophic fungal plant pathogen and two plant phytohormones (salicylic acid and methyl jasmonate) was investigated using *Agrobacterium*-mediated transient expression on two tobacco species. A β-glucuronidase gene with intron was used in each construct as a reporter gene.

The basal expression level is a crucial factor which defines the expression of a transgene before pathogen invasion and it is considered as an undesirable characteristic for an inducible promoter (Gurr and Rushton 2005; Rushton et al. 2002). In the previous study we have shown SP-FF did not have basal expression in the stable transformed canola plants (Shokouhifar et al. 2011b). It has already been shown that, the transformed *Arabidopsis* by a synthetic promoter containing a tetramer of D elements had no appreciable background expression in any part of this plant (Rushton et al. 2002). The outcomes of the recent study affirmed that, the basal expression of SP-FF and SP-DD compared to pGCGi as a constitutive promoter was not macroscopically visible. Moreover, no considerable basal expression was observed regarding to SP-FFDD as a heterotetramer which contained a combination of F and D elements.

The response of tobacco species (*N. tabacum* cv. Xanthi and *Nicotiana benthamiana*) which were transiently harboring the dimmer forms of D, to salicylic acid treatment was more than methyl jasmonate. Furthermore, there was a little sensitivity to the promoter containing two repetitions of D element in both *A. rabiei*’s pathotypes which is suggesting that, D element is much more inducible by salicylic acid than jasmonic acid signaling pathway as well as the necrotrophic pathogens like *A. rabiei*. Conversely, higher rate of expression and inducibility for methyl jasmonate and *A. rabiei* elicitors’ were occurred in tobacco plants which were transiently harboring the dimmer forms of F element. A recent study showed that, D element expressed the reporter gene 24 h after treatment by salicylic acid. This is consistent with the previous reports that claimed a synthetic promoter contains D tetramer was responsive to compatible and incompatible pathosystems (Rushton et al. 2002). The signaling pathway mediated by salicylic acid are associated with hypersensitive reaction (HR) as well as cell programmed death and they are able to trigger resistance against biotrophic and hemi-biotrophic pathogens (Glazebrook 2005; Robert-Seilaniantz et al. 2011). Our results indicated that, SP-FF was not induced in response to salicylic acid treatment in a transient expression system in tobacco plants. It has previously been reported that, transgenic canola plants harboring SP-FF was not significantly induced by salicylic acid treatment (Shokouhifar et al. 2011a). The F element classified as a unique member of W-box *cis*-acting elements that are responsive to WRKY transcription factors (Heise et al. 2002). Although F element as a type of W box *cis*-acting elements is expected to be inducible by salicylic acid (Yang et al. 1999), but having an additional 9 bp motif caused its different inducibility (Heise et al. 2002).

In the present study, we have combined two *cis*-acting elements; F and D in SP-FFDD in order to collect their advantages in a unique promoter. Transient GUS assay revealed that, SP-FFDD is responsive to salicylic acid treatment. This indicates that, D element had a dominant effect on F element. Recently a similar synthetic promoter named SP-DDEE was constructed whose possessed a combination of D and E elements (Kirsch et al. 2000). SP-DDEE is able to express a chitinase gene which was effectively assayed against *Sclerotinia sclerotiorum* and *Rhizoctonia solani* in transgenic canola plants (Moradyar et al. 2016).

Our first expression assay showed reaction of D and F elements in response to salicylic acid treatment. Salicylic acid is a well-known defense signaling node involved in triggering of resistant against biotrophic pathogens (Glazebrook 2005; Robert-Seilaniantz et al. 2011). To show the expression pattern of the synthetic promoters in response to necrotrophic pathogens we assayed their response to methyl jasmonate treatment. Methyl jasmonate is known as a plant signaling molecule involved in some developmental and defending aspects in plant biology. The methyl jasmonate pathway is responsible for triggering resistance against necrotrophic pathogens (Jones and Dangl 2006). These pathogens utilize a usual virulence approach that involves in rapid killing of plant cells to gain nutrients (Antico et al. 2012; Glazebrook 2005; Kunkel and Brooks 2002; Pozo et al. 2004). We used the dimer form of F element because of its strong reactivity against fungal infection and not wounding. Furthermore, the monomer form of the F element has low sensitivity against fungal contamination (Heise et al. 2002). Our results showed that, the dimmer form of F element in constructed SP-FF promoter was induced by methyl jasmonate but by salicylic acid. This is in agreement with a research shown that, transgenic canola harboring the F element was considerably induced by methyl jasmonate (Shokouhifar et al. 2011a). In addition, two copies of D *cis*-acting element were less responsive to methyl jasmonate than that of two copies of F element. D element was also responsive to salicylic acid treatment. Recent evidence has shown that, the prompt activation of jasmonic acid-mediated response by certain species of biotrophic fungi. This may suggest kind of relationship between salicylic acid and jasmonic acid mediated pathways (Antico et al. 2012). We also tested the effect of methyl jasmonate treatment on SP-FFDD promoter. This promoter showed less activation to methyl jasmonate treatment than SP-FF. As two copies of F element together with two copies of D element were included in this promoter, the probable dominant effect of D element on F element may causes little responsiveness for this construct to methyl jasmonate treatment compared to SP-FF promoter.

*A. rabiei* is a necrotrophic fungal plant pathogen which causes the blighted spots on the leaves, buds and even stems of Chickpea (Kaiser 1973). The fungus can still penetrate to the pod and infect the seed. Multiple cycles of infection can occur during the growing season and cause severe damage to the crop.

Jasmonates like Jasmonic acid and methyl jasmonate (plant hormone regulators) stimulate proteinase inhibitor proteins in response to necrotrophic pathogens and thus limits the fungal growth (Antico et al. 2012; Gfeller et al. 2010). In different studies, it has been shown that exogenous application of methyl jasmonate increases resistance against a number of necrotrophic fungal species. For examples; the pretreated wheat (*Triticum aestivum*) by methyl jasmonate showed a delayed developmental symptom against *Fusarium pseudograminearum* (Desmond et al. 2006) and enhanced resistance to the infection made by *Stagonospora nodorum* (Jayaraj et al. 2004). Concerning to the application of jasmonate and ethylene in plants, a report showed that, these phytohormones were able to enhance maize resistance against a number of necrotrophic pathogens like *Rhizopus microspores* and *Colletotrichum graminicola* (Schmelz et al. 2011).

In this study, we have investigated the application of necrotrophic fungus *A. rabiei* ASR009 elicitor probably mediated by jasmonic acid (or its derivative methyl jasmonate) signaling pathway in tobacco plants and triggered the activation of SP-FF promoter and subsequently induced the expression of GUS gene. The external application of methyl jasmonate showed the same result as application of *A. rabiei* ASR009 elicitor. No visible difference was detected between *N. tabacum* cv. Xanthi and *Nicotiana benthamiana* plants. *A. rabiei* ASR003 elicitor was also prompted the activation of SP-FF promoter but this was less by ASR009 elicitor in all repetitions. SP-FFDD promoter showed a medium inducibility in both ASR003 and ASR009 elicitors. SP-DD promoter was a little inducible for this pathogen. It may be applicable to use SP-FF and SP-FFDD synthetic promoters in *A. rabiei* and other necrotrophic pathogens in the resistance programs in future. More studies must be performed regarding to SP-DD promoter due to the complexity of D box in response to both biotrophic and necrotrophic pathogens.

## Supplementary information

**Additional file 1. Figure S1.** Effects of salicylic acid treatment on pGCGi, pGDD, pGFF and pGFFDDconstructs evaluated on two tobacco species. **Figure S2**. Effect of methyl jasmonate treatment on pGCGi, pGDD, pGFF and pGFFDD constructs evaluated on two tobacco species. **Figure S3**. Effect of *Ascochyta rabiei* pathotype ASR009 on pGCGi, pGDD, pGFF and pGFFDD constructs evaluated on two tobacco species. **Figure S4**. Effect of *Ascochyta rabiei* pathotype ASR003 on pGCGi, pGDD, pGFF and pGFFDD constructs evaluated on two tobacco species.

## Data Availability

All data are presented in figures and tables within this article. Any material used in this study will be available for research purposes upon request.

## Abbreviations

SP-DD: synthetic promoter-D box-D Box
SP-FF: synthetic promoter-F element-F element
SP-FFDD: synthetic promoter-F element-F element-D box-D Box
ASR003: *Ascochyta rabiei isolate number* 003
ASR009: *Ascochyta rabiei isolate number* 009
GUS: β-glocronidase
WRKY: a group of transcription factors
PCR: polymerase chain reaction
