# Supplementary material for: Transient expression analysis of synthetic promoters containing F and D cis-acting elements in response to Ascochyta rabiei and two plant defense hormones
Source: AMB Express. 2019 Dec 4;9:195. doi: 10.1186/s13568-019-0919-x (PMC6892989; doi:10.1186/s13568-019-0919-x)
Supplement: Supplementary file 1 — Additional file 1. Figure S1. Effects of salicylic acid treatment on pGCGi, pGDD, pGFF and pGFFDDconstructs evaluated on two tobacco species. Figure S2. Effect of methyl jasmonate treatment on pGCGi, pGDD, pGFF and pGFFDD constructs evaluated on two tobacco species. Figure S3. Effect of Ascochyta rabiei pathotype ASR009 on pGCGi, pGDD, pGFF and pGFFDD constructs evaluated on two tobacco species. Figure S4. Effect of Ascochyta rabiei pathotype ASR003 on pGCGi, pGDD, pGFF and pGFFDD constructs evaluated on two tobacco species. [file 13568_2019_919_MOESM1_ESM.docx]

| pGCGi | Control (*N. tabacum* cv Xanthi)  1 2 3 | | | Salicylic acid (*N.* *tabacum* cv Xanthi)  1 2 3 | | |
| --- | --- | --- | --- | --- | --- | --- |
|  | 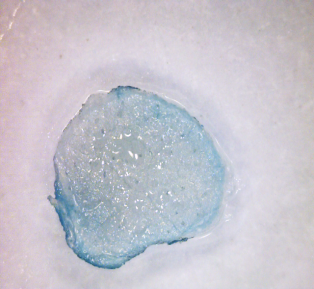 | 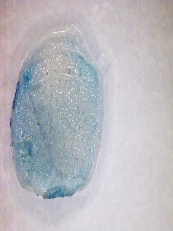 | 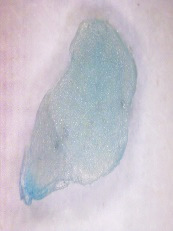 | 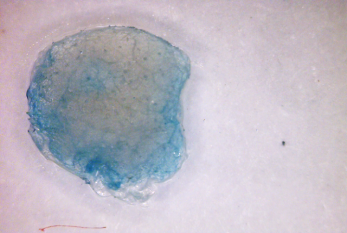 | 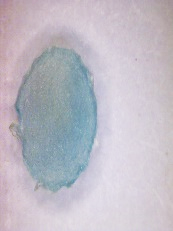 | 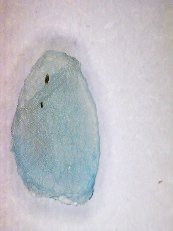 |
|  | Control (*N. benthamiana*)  1 2 3 | | | Salicylic acid (*N. benthamiana*)  1 2 3 | | |
|  | 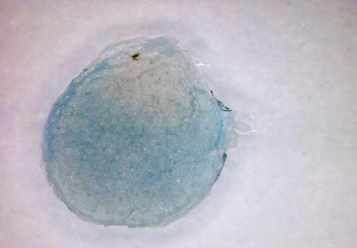 | 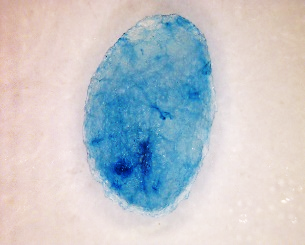 | 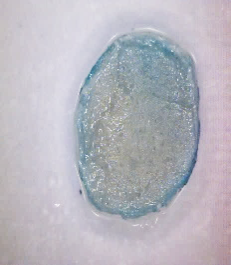 | 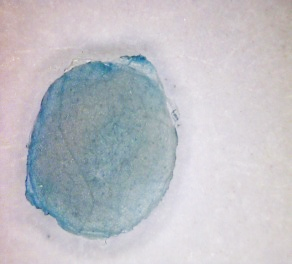 | 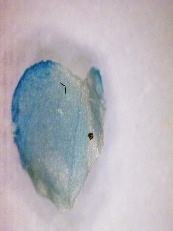 | 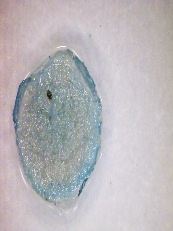 |
| pGDD | Control (*N. tabacum* cv Xanthi)  1 2 3 | | | Salicylic acid (*N.tabacum* cv Xanthi)  1 2 3 | | |
|  | 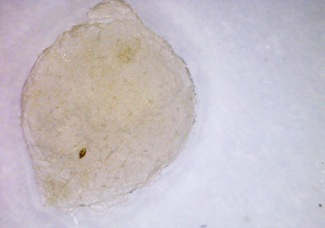 | 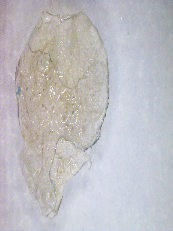 | 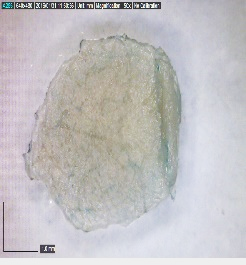 | 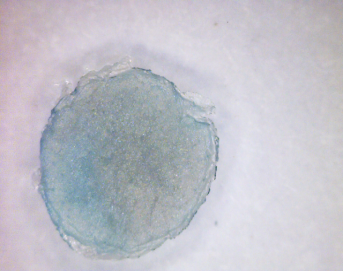 | 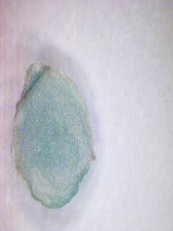 | 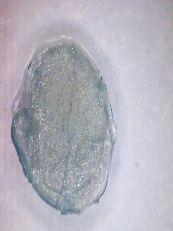 |
|  | Control (*N. benthamiana*)  1 2 3 | | | Salicylic acid (*N. benthamiana*)  1 2 3 | | |
|  | 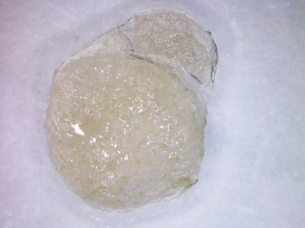 | 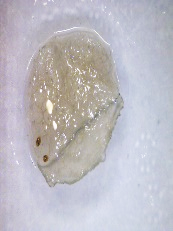 | 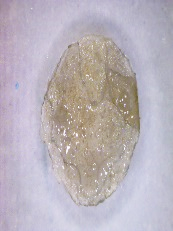 | 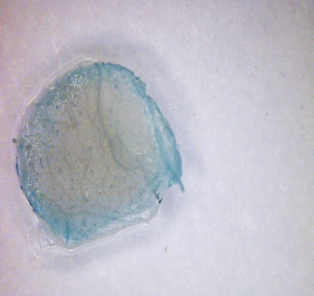 | 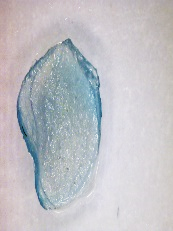 | 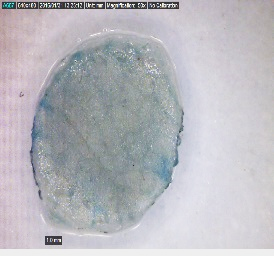 |
| pGFF | Control (*N. tabacum* cv Xanthi)  1 2 3 | | | Salicylic acid (*N.* *tabacum* cv Xanthi)  1 2 3 | | |
|  | 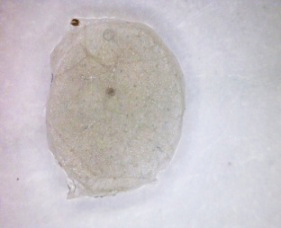 | 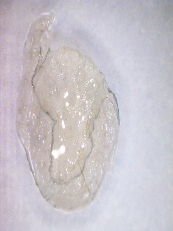 | 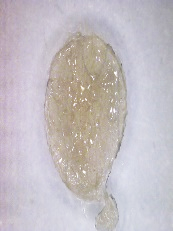 | 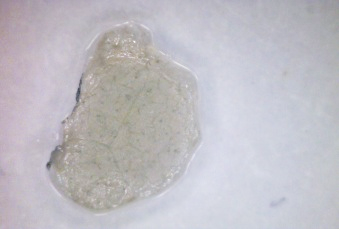 | 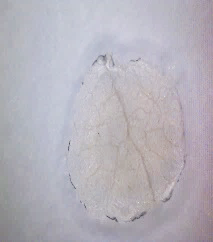 | 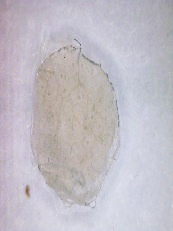 |
|  | Control (*N. benthamiana*)  1 2 3 | | | Salicylic acid (*N. benthamiana*)  1 2 3 | | |
|  | 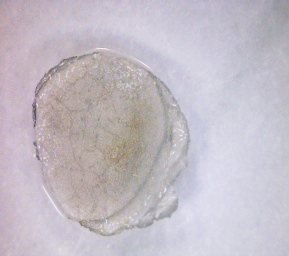 | 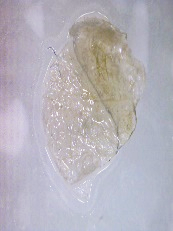 | 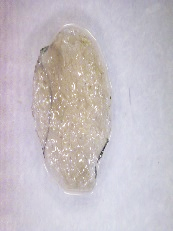 | 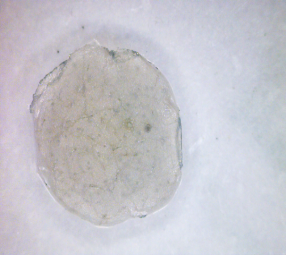 | 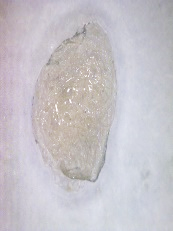 | 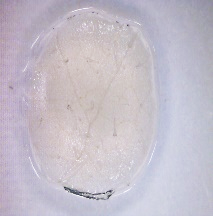 |
| pGFFDD | Control (*N. tabacum* cv Xanthi)  1 2 3 | | | Salicylic acid (*N.* *tabacum* cv Xanthi)  1 2 3 | | |
|  | 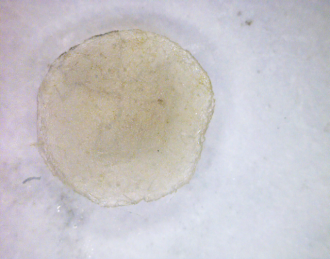 | 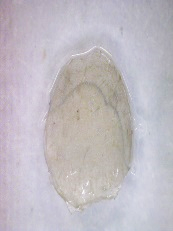 | 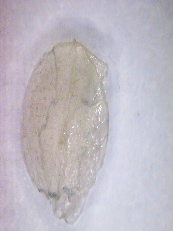 | 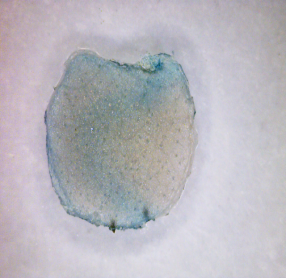 | 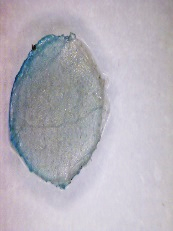 | 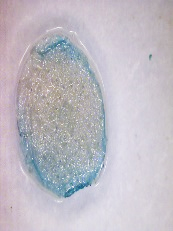 |
|  | Control (*N. benthamiana*)  1 2 3 | | | Salicylic acid (*N. benthamiana*)  1 2 3 | | |
|  | 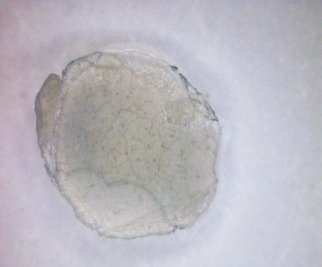 | 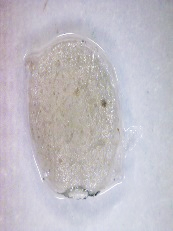 | 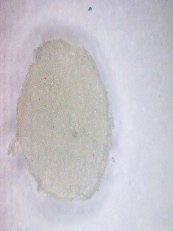 | 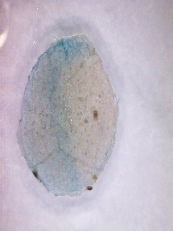 | 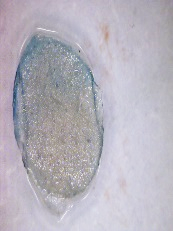 | 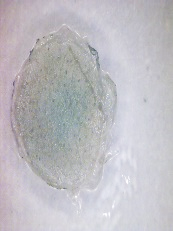 |

**Figure S1.** Effects of salicylic acid treatment on pGCGi, pGDD, pGFF and pGFFDD constructs evaluated on two tobacco species; *N. tabacum* cv. Xanthi and *N. benthamiana*. Agro-injected plants without salicylic acid treatment were used as control. Three replications (as shown; 1, 2 and 3) have been provided for each treatment and its related control.

| pGCGi | Control (*N. tabacum* cv Xanthi)  1 2 3 | | | Methyl jasmonate (*N.* *tabacum* cv Xanthi)  1 2 3 | | |
| --- | --- | --- | --- | --- | --- | --- |
|  | 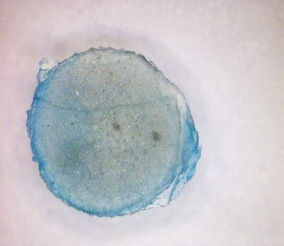 | 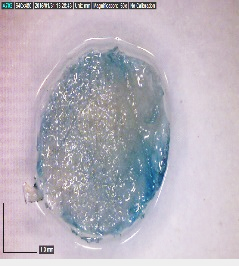 | 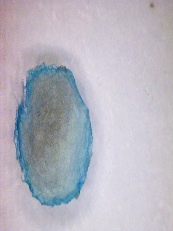 | 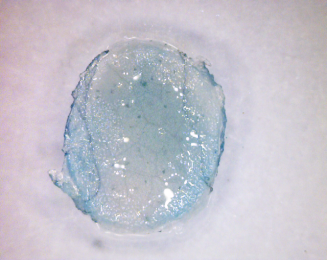 | 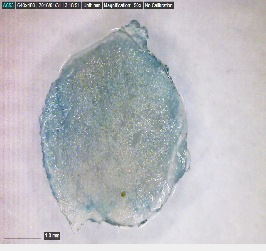 | 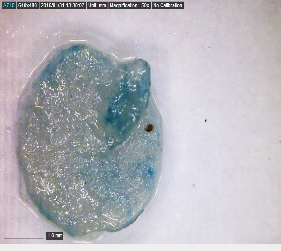 |
|  | Control (*N. benthamiana*)  1 2 3 | | | Methyl jasmonate (*N. benthamiana*)  1 2 3 | | |
|  | 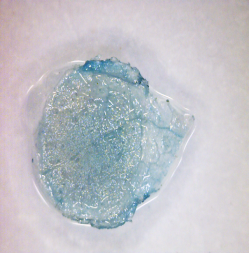 | 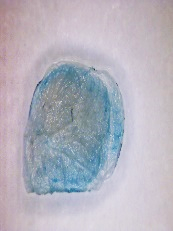 | 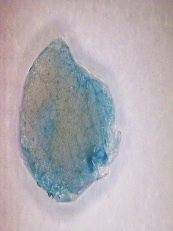 | 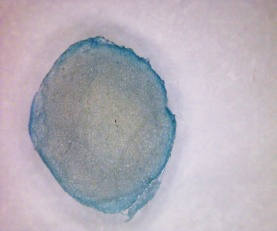 | 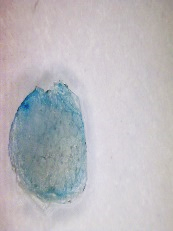 | 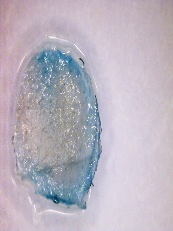 |
| pGDD | Control (*N. tabacum* cv Xanthi)  1 2 3 | | | Methyl jasmonate (*N.* *tabacum* cv Xanthi)  1 2 3 | | |
|  | 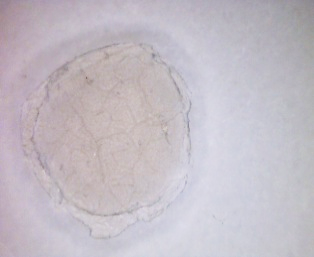 | 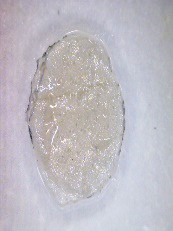 | 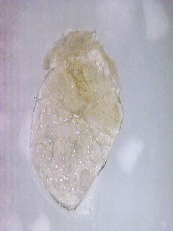 | 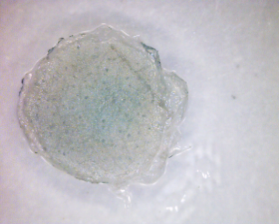 | 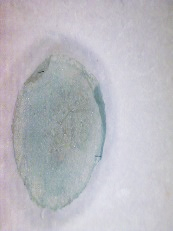 | 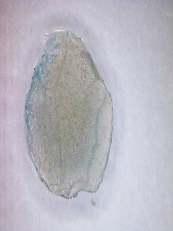 |
|  | Control (*N. benthamiana*)  1 2 3 | | | Methyl jasmonate (*N. benthamiana*)  1 2 3 | | |
|  | 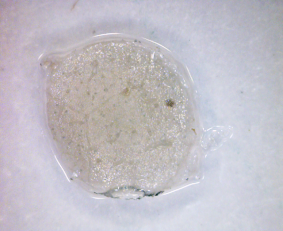 | 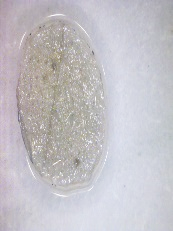 | 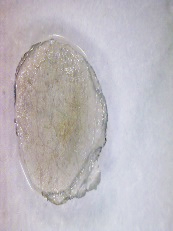 | 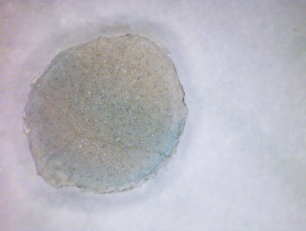 | 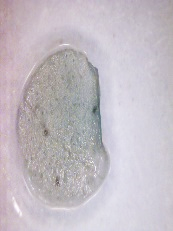 | 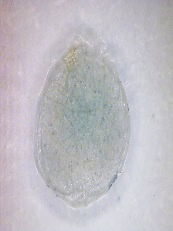 |
| pGFF | Control (*N. tabacum* cv Xanthi)  1 2 3 | | | Methyl jasmonate (*N.* *tabacum* cv Xanthi)  1 2 3 | | |
|  | 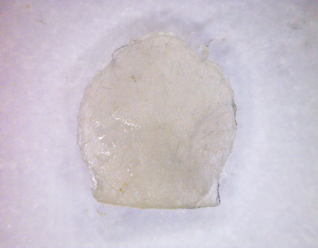 | 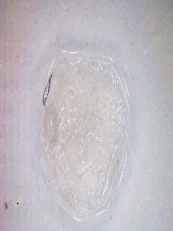 | 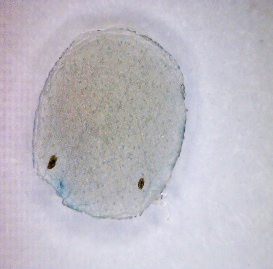 | 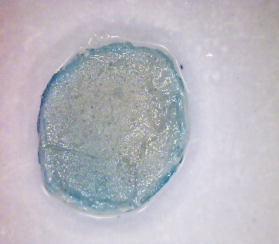 | 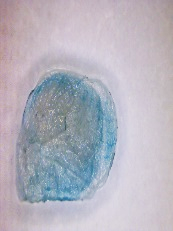 | 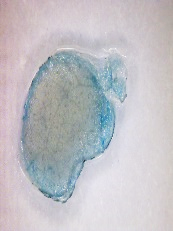 |
|  | Control (*N. benthamiana*)  1 2 3 | | | Methyl jasmonate (*N. benthamiana*)  1 2 3 | | |
|  | 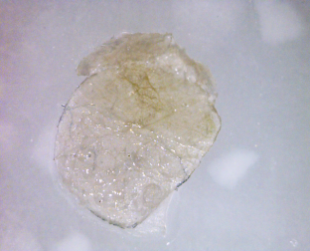 | 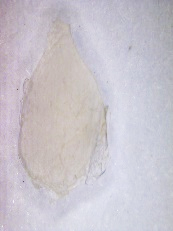 | 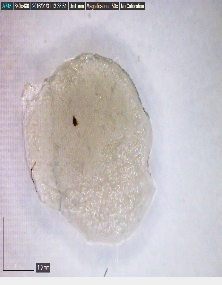 | 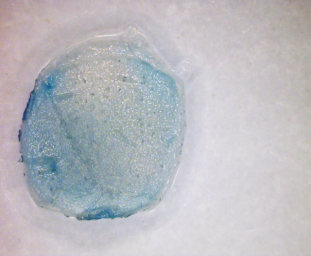 | 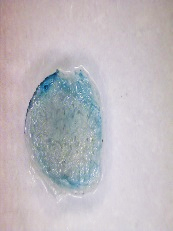 | 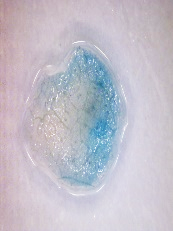 |
| pGFFDD | Control (*N. tabacum* cv Xanthi)  1 2 3 | | | Methyl jasmonate (*N.* *tabacum* cv Xanthi)  1 2 3 | | |
|  | 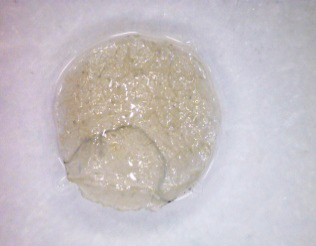 | 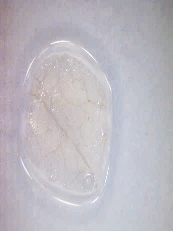 | 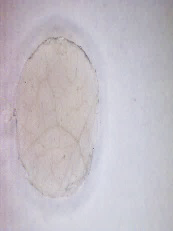 | 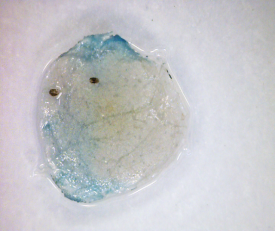 | 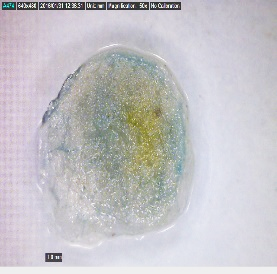 | 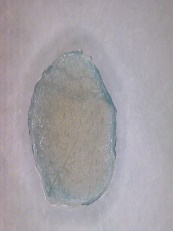 |
|  | Control (*N. benthamiana*)  1 2 3 | | | Methyl jasmonate (*N. benthamiana*)  1 2 3 | | |
|  | 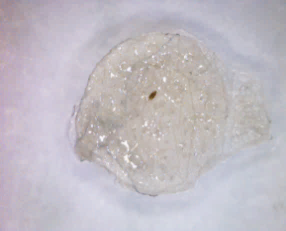 | 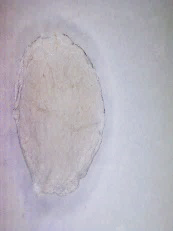 | 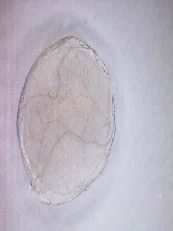 | 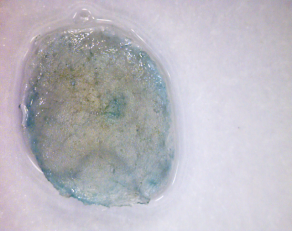 | 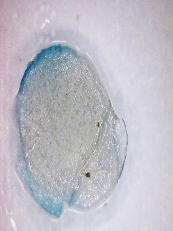 | 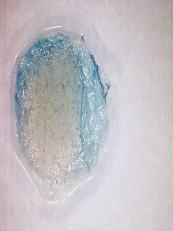 |

**Figure S2.** Effect of methyl jasmonate treatment on pGCGi, pGDD, pGFF and pGFFDD constructs evaluated on two tobacco species; *N. tabacum* cv. Xanthi and *N. benthamiana*. Agro-injected plants without methyl jasmonate treatment were used as control. Three replications (as shown; 1, 2 and 3) have been provided for each treatment and its related control.

| pGCGi | Control (*N. tabacum* cv Xanthi)  1 2 3 | | | ASR009 (*N.* *tabacum* cv Xanthi)  1 2 3 | | |
| --- | --- | --- | --- | --- | --- | --- |
|  | 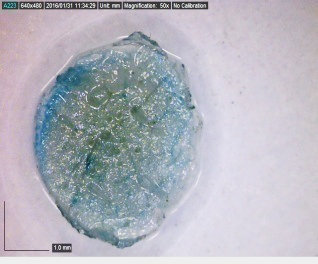 | 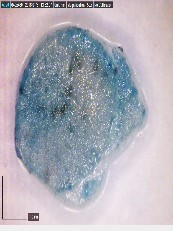 | 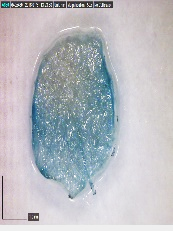 | 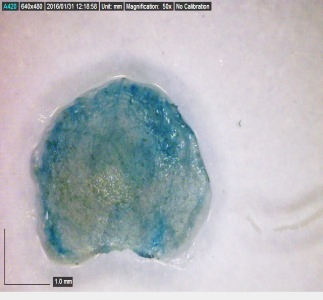 |  |  |
|  | Control (*N. benthamiana*)  1 2 3 | | | ASR009 (*N. benthamiana*)  1 2 3 | | |
|  |  |  |  |  |  |  |
| pGDD | Control (*N. tabacum* cv Xanthi)  1 2 3 | | | ASR009 (*N.* *tabacum* cv Xanthi)  1 2 3 | | |
|  |  |  |  |  |  |  |
|  | Control (*N. benthamiana*)  1 2 3 | | | ASR009 (*N. benthamiana*)  1 2 3 | | |
|  |  |  |  |  |  |  |
| pGFF | Control (*N. tabacum* cv Xanthi)  1 2 3 | | | ASR009 (*N.* *tabacum* cv Xanthi)  1 2 3 | | |
|  |  |  |  |  |  |  |
|  | Control (*N. benthamiana*)  1 2 3 | | | ASR009 (*N. benthamiana*)  1 2 3 | | |
|  |  |  |  |  |  |  |
| pGFFDD | Control (*N. tabacum* cv Xanthi)  1 2 3 | | | ASR009 (*N.* *tabacum* cv Xanthi)  1 2 3 | | |
|  |  |  |  |  |  |  |
|  | Control (*N. benthamiana*)  1 2 3 | | | ASR009 (*N. benthamiana*)  1 2 3 | | |
|  |  |  |  |  |  |  |

**Figure S3.** Effect of *Ascochyta rabiei* pathotype ASR009 on pGCGi, pGDD, pGFF and pGFFDD constructs evaluated on two tobacco species; *N. tabacum* cv. Xanthi and *N. benthamiana*. Agro-injected plants without treatment by fungal elicitor used as control. Three replications (as shown; 1, 2 and 3) have been provided for each treatment and its related control.

| pGCGi | Control (*N. tabacum* cv Xanthi)  1 2 3 | | | ASR003 (*N.* *tabacum* cv Xanthi)  1 2 3 | | |
| --- | --- | --- | --- | --- | --- | --- |
|  |  |  |  |  |  |  |
|  | Control (*N. benthamiana*)  1 2 3 | | | ASR003 (*N. benthamiana*)  1 2 3 | | |
|  |  |  |  |  |  |  |
| pGDD | Control (*N. tabacum* cv Xanthi)  1 2 3 | | | ASR003 (*N.* *tabacum* cv Xanthi)  1 2 3 | | |
|  |  |  |  |  |  |  |
|  | Control (*N. benthamiana*)  1 2 3 | | | ASR003 (*N. benthamiana*)  1 2 3 | | |
|  |  |  |  |  |  |  |
| pGFF | Control (*N. tabacum* cv Xanthi)  1 2 3 | | | ASR003 (*N.* *tabacum* cv Xanthi)  1 2 3 | | |
|  |  |  |  |  |  |  |
|  | Control (*N. benthamiana*)  1 2 3 | | | ASR003 (*N. benthamiana*)  1 2 3 | | |
|  |  |  |  |  |  |  |
| pGFFDD | Control (*N. tabacum* cv Xanthi)  1 2 3 | | | ASR003 (*N.* *tabacum* cv Xanthi)  1 2 3 | | |
|  |  |  |  |  |  |  |
|  | Control (*N. benthamiana*)  1 2 3 | | | ASR003 (*N. benthamiana*)  1 2 3 | | |
|  |  |  |  |  |  |  |

**Figure S4.** Effect of *Ascochyta rabiei* pathotype ASR003 on pGCGi, pGDD, pGFF and pGFFDD constructs evaluated on two tobacco species; *N. tabacum* cv. Xanthi and *N. benthamiana*. Agro-injected plants without treatment by fungal elicitor used as control. Three replications (as shown; 1, 2 and 3) have been provided for each treatment and its related control.
